# Supplementary material for: The Construction and Exploration of a Comprehensive MicroRNA Centered Regulatory Network in Foxtail Millet (Setaria italica L.)
Source: Front Plant Sci. 2022 May 6;13:848474. doi: 10.3389/fpls.2022.848474 (PMC9121102; doi:10.3389/fpls.2022.848474)
Supplement: Supplementary file 1 [file Data_Sheet_1.zip › Sit-miRN3020_Seita.5G110100.1_175_TPlot.pdf]

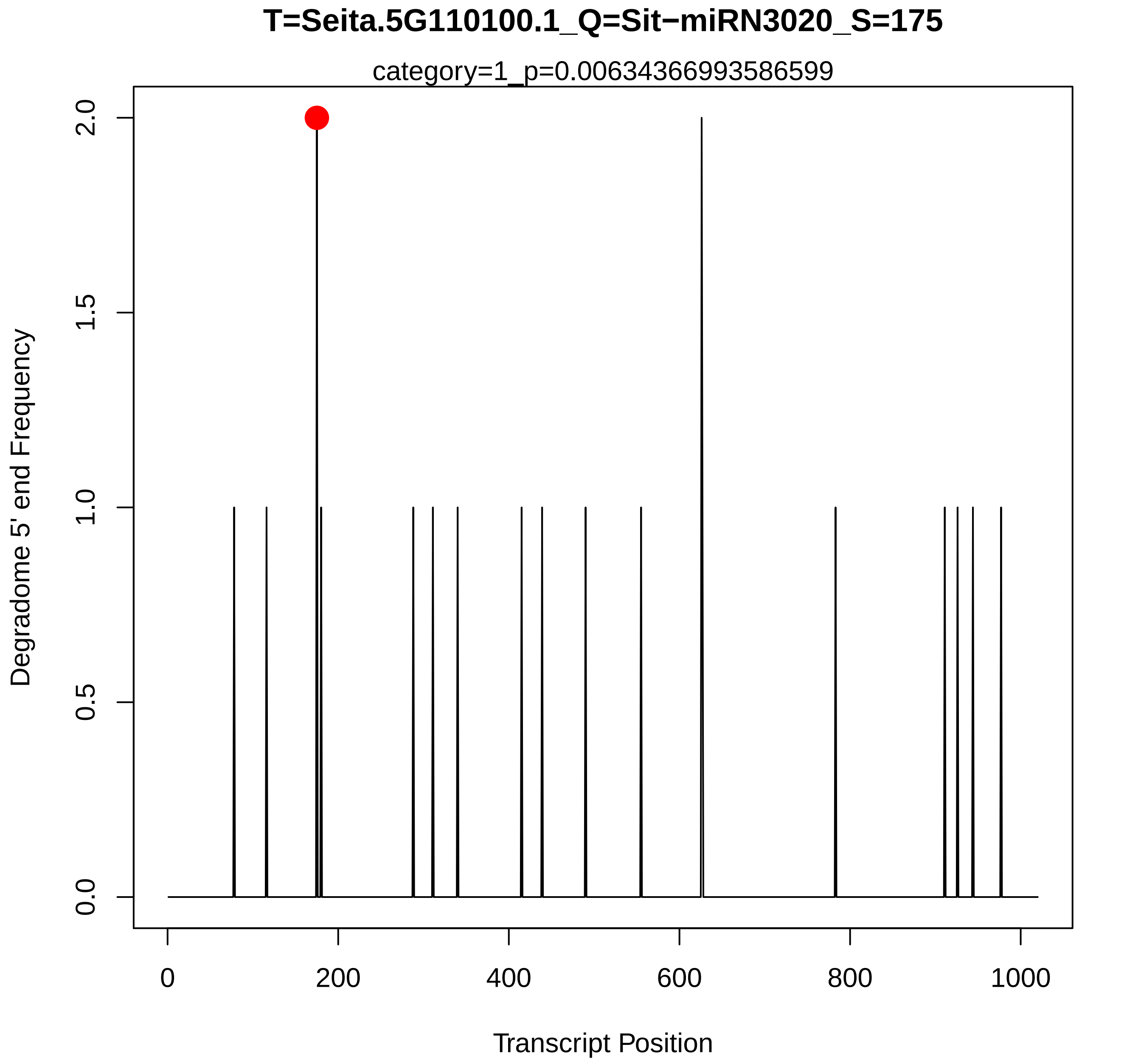
T=Seita.5G110100.1_Q=Sit−miRN3020_S=175
category=1_p=0.00634366993586599
Degradome 5' end Frequency
0.0
0.5
1.0
1.5
2.0
0
200
400
600
800
1000
Transcript Position
